# A systematic review of brain metastases from lung cancer using magnetic resonance neuroimaging: Clinical and technical aspects

**DOI:** 10.1002/jmrs.756

**Published:** 2024-01-18

**Authors:** Sadegh Ghaderi, Sana Mohammadi, Mahdi Mohammadi, Zahra Najafi Asli Pashaki, Mehrsa Heidari, Rahim Khatyal, Rasa Zafari

**Affiliations:** ^1^ Department of Neuroscience and Addiction Studies, School of Advanced Technologies in Medicine Tehran University of Medical Sciences Tehran Iran; ^2^ Department of Medical Sciences, School of Medicine Iran University of Medical Sciences Tehran Iran; ^3^ Department of Medical Physics and Biomedical Engineering, School of Medicine Tehran University of Medical Sciences Tehran Iran; ^4^ Department of Medical Physics, School of Medicine Iran University of Medical Sciences Tehran Iran; ^5^ Department of Medical Science, School of Medicine Ahvaz Jundishapur University of Medical Sciences Ahvaz Iran; ^6^ Department of Radiology, Faculty of Allied Medical Sciences Tabriz University of Medical Sciences Tabriz Iran; ^7^ School of Medicine Tehran University of Medical Sciences Tehran Iran

**Keywords:** Brain metastases, deep learning, lung cancer, MRI, radiomics

## Abstract

**Introduction:**

Brain metastases (BMs) are common in lung cancer (LC) and are associated with poor prognosis. Magnetic resonance imaging (MRI) plays a vital role in the detection, diagnosis and management of BMs. This review summarises recent advances in MRI techniques for BMs from LC.

**Methods:**

This systematic review was conducted following the Preferred Reporting Items for Systematic Reviews and Meta‐Analyses (PRISMA) guidelines. A comprehensive literature search was conducted in three electronic databases: PubMed, Scopus and the Web of Science. The search was limited to studies published between January 2000 and March 2023. The quality of the included studies was evaluated using appropriate tools for different study designs. A narrative synthesis was carried out to describe the key findings of the included studies.

**Results:**

Sixty‐five studies were included. Standard MRI sequences such as T1‐weighted (T1w), T2‐weighted (T2w) and fluid‐attenuated inversion recovery (FLAIR) were commonly used. Advanced techniques included perfusion‐weighted imaging (PWI), diffusion‐weighted imaging (DWI) and radiomics analysis. DWI and PWI parameters could distinguish tumour recurrence from radiation necrosis. Radiomics models predicted genetic mutations and the risk of BMs. Diagnostic accuracy was improved with deep learning (DL) approaches. Prognostic factors such as performance status and concurrent chemotherapy impacted survival.

**Conclusion:**

Advanced MRI techniques and specialised MRI methods have emerging roles in managing BMs from LC. PWI and DWI improve diagnostic accuracy in treated BMs. Radiomics and DL facilitate personalised prognosis and treatment. Magnetic resonance imaging plays a key role in the continuum of care for BMs of patients with LC, from screening to treatment monitoring.

## Graphical abstract

In this systematic review, we explore the latest advancements in MRI techniques used to detect and characterise brain metastases in lung cancer patients. We focus on MRI and machine learning methods, which have shown potential in enhancing diagnosis, prognosis and personalised treatment for those with lung cancer and brain metastases.

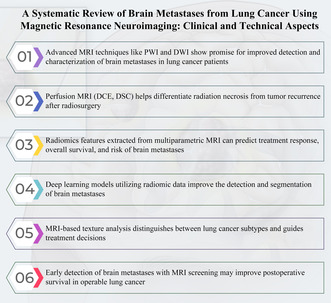

## Introduction

Brain metastases (BMs) are a common complication of cancer, affecting up to 40% of patients at some point during the course of the disease.^1^ Brain metastases are the most common type of cancer metastasis and, as secondary brain neoplasms, are the most common type of intracranial tumour in adults (20%–40%).^2^, ^3^ Sixty‐seven to 80 % of BMs originate primarily from lung cancer (LC), breast cancer and melanoma.^4^ In patients with BMs, early diagnosis has an essential role in the maintenance of motor functions.^3^

Magnetic resonance imaging (MRI) has excellent cerebral soft‐tissue discrimination and the range of sequences can explore differences in the biophysical properties of the brain and tumours.^3^ Traditionally, contrast‐enhanced (CE) MRI is the preferred imaging study for the diagnosis of BMs.^3^ The two most commonly used MRI sequences for assessing BMs are CE T1‐weighted (T1‐w) and T2‐weighted fluid‐attenuated inversion recovery (T2‐w FLAIR), which provide information about size, morphology and macroscopic structures.^3^ Advancements in MRI technology now enable the modality to detect metastases that may not be visible using standard image acquisition protocols.^5^ Therefore, MRI plays a vital role in diagnosing, determining the most effective treatment plan, monitoring response to therapy and even predicting prognosis.^6^

Management of BMs has traditionally involved a combination of surgery, radiation therapy and systemic therapy.^7^, ^8^ Early diagnosis as a result of more precise and innovative neuroimaging modalities^9^ affects prognosis and outcome^10^; besides, the ability to correctly diagnose tumour types is necessary before treatment initiation.^11^ Therefore, the detection of BM is important for the initial staging of patients with LC.^12^

Magnetic resonance imaging is more sensitive than computed tomography (CT) scans for early detection of recurrence, allowing for earlier treatment and better outcomes.^3^, ^13^ Furthermore, advances in MRI technology have led to the development of specialised MRI techniques that can be used to improve the diagnosis, treatment and monitoring of BMs.^14^ Advanced MRI techniques can detect small brain lesions such as metastases, determine size and location, and assess blood supply to guide treatment decisions. More recently, these techniques have moved beyond anatomical imaging to enable the characterisation of microstructures, cellularity, physiology and metabolism (such as diffusion‐weighted imaging (DWI), susceptibility‐weighted imaging (SWI), perfusion‐weighted imaging (PWI), and magnetic resonance spectroscopy (MRS)).^15^

Recent advances in radiomics and deep learning (DL) applied to MRI data have shown promise in improving diagnostic, prognostic and predictive capabilities.^16^ Radiomics extracts quantitative imaging features from MRI scans that can reveal information about the tumour phenotype, while DL methods (DLMs) can analyse raw imaging data directly to make inferences.^17^, ^18^ By applying these techniques to MRI data from BMs of patients with LC, we may gain new insights into tumour biology, treatment response and clinical outcomes.^19^

However, the available literature on the role of MRI in the management of BMs from LC is heterogeneous and lacks a comprehensive evaluation. Therefore, the present systematic review aims to investigate the role of MRI techniques in the diagnosis and management of BMs from LC with a particular focus on clinical and technical aspects.

## Methods

### Search strategy and study selection

This systematic review was conducted following the Preferred Reporting Items for Systematic Reviews and Meta‐Analyses (PRISMA) guidelines.^20^ A comprehensive literature search was conducted in three electronic databases: PubMed, Scopus and Web of Science. The search strategy included the following keywords and MeSH terms: ‘brain metastases’, ‘MRI’ and ‘lung cancer’. The search was limited to studies published between January 2000 and March 2023. Reference lists of eligible studies and relevant reviews were also manually searched for additional citations. The search query for each database was as follows: ((brain metastases [Title/Abstract]) AND (MRI [Title/Abstract])) AND (lung cancer [Title/Abstract]).

Two independent reviewers screened the titles and abstracts of the identified records using predefined eligibility criteria. Disagreements between reviewers (S.Gh. and M.M.) were resolved through discussion or by consulting a third reviewer (S.M.). The eligibility criteria for inclusion in this study included research investigations specifically centred on BMs occurring in patients with LC, including studies that employed MRI for diagnostic or treatment purposes. The study also restricted the publication to those in the English language. It sought to incorporate a broad spectrum of original research articles, including observational, experimental and clinical trial designs. Additionally, non‐original research formats such as review articles, case reports, letters, editorials and conference abstracts were considered.

### Data extraction and quality assessment

Data extraction was performed independently by two reviewers using a standardised data collection form (S.Gh and S.M). Data extracted included study characteristics, patient population and outcomes of interest. The methodological quality of the included studies was assessed using the appropriate tools for different study designs. For example, the Newcastle–Ottawa Scale (NOS)^21^ was used for assessing the quality of observational studies, and the Cochrane Collaboration's RoB 2.0 tool^22^ was used for randomised controlled trials. A narrative synthesis was conducted to describe the key findings of the included studies. Due to the heterogeneity in study designs, populations and outcome measures, a meta‐analysis was not conducted. The results were organised according to the main themes identified during the data extraction process (Table 1).

**Table 1:** MRI and clinical findings of brain metastases from lung cancer

Publication	Subjects (Male/Female)/Mean Age (years)	Tumoral properties	MRI techniques	Technical Findings	Clinical Findings
Hochstenba et al. 2000^54^	125	SCLC	T1‐w, T1‐w post, PD‐w, and T2‐w	Progression of BM	Median survival time for patients with BMs was 9.5 months. No significant difference in survival between symptomatic or asymptomatic BMs and limited disease patients.
Kim et al. 2005^55^	183 (139/44)/67	NSCLC	T1‐w and T1‐w post	Limited brain MRI (T1‐w and T1‐w post) is viable for detecting BMs. Cost‐effective method for detecting BMs at initial LC staging.	Median survival duration for the trial group: 43 weeks. One‐year survival rate for the trial group: 39.5%. Median survival duration for the control group: 31 weeks. One‐year survival rate for the control group: 16.7%.
Park et al. 2007^25^	83 (41/42)	Adenocarcinoma and bronchioloalveolar carcinoma	T1‐w, T1‐w post, and T2‐w	Pre‐operative MR screening of the brain may increase survival in operable lung adenocarcinoma. Screening for BMs before surgery may improve postoperative survival in lung adenocarcinoma patients.	57% of patients experienced recurrence after primary lung adenocarcinoma resection.
Takeda et al. 2008^56^	53	LC	T1‐w 2D SE, T1‐w post 3D MPRAGE, T1‐w post 2D SE, T2‐w 2D SE	3D MPRAGE detected BMs more effectively than 2D SE.	NA
Zhang et al. 2009^57^	45	LC	T1‐w, T1‐w post, T2‐w, and SWI	SWI is essential in diagnosing most microbleeds. LC metastasis can cause brain bleeding. SWI detects bleeding in the majority of patients.	NA
Hakyemez et al. 2010^58^	18	LC	T1‐w, T2‐w, FLAIR, and DSC	Solitary masses differentiation: Mass and rCBV ratios of edema around the tumour are helpful. Distinguishing metastases from high‐grade gliomas. Before surgery.	NA
Yang et al. 2011^59^	31	NSCLC and SCLC	T1‐w post and T2‐w	MRI‐extracted tumour volume is a superior indicator of maximum diameter. It allows for a more accurate assessment of potential radiotherapy techniques for metastatic brain tumours.	Clinical follow‐up after SRS: 51% reported symptom improvement. 23% reported stable symptoms. 18% experienced progression. Corticosteroid usage: 54% reduced dosage or discontinued use Side effects: 13% showed signs of increasing intracranial pressure 7 cases required craniotomy
Szerlip et al. 2011^60^	21	NSCLC and SCLC	T1‐w, T1‐w post, T2‐w, and T2‐w FLAIR	Volumetric WM changes are measured after WBRT This measurement is used to assess cognitive and functional decline	Median survival time after WBRT: 24 months. Two‐year survival probability: 45%. Three‐year survival probability: 11%.
Loganathan et al. 2012^61^	85	LC	T1‐w FSPGR	GKRS treatment plans with 3 T MRI did not affect: Likelihood of distant brain failure. Need for WBRT. Neurological death.	No significant difference in OS. No significant difference in the likelihood of neurological death.
Ono et al. 2013^37^	1792	LC	T1‐w, T1‐w post, T2‐w, T2‐w FLAIR, and DWI	Punctate/weak ring enhancement without perifocal edema = early‐stage metastases of SCLC. Double/triple‐ring enhancement with back‐and‐forth high/low intensity layers = characteristic of SCLC.	NA
Jakubovic et al. 2014^62^	12	LC	T1‐w post‐SPGR, T1‐w FLAIR, FLAIR, DCE, and DSC	Lower K^2trans^ at 1 week. Lower rCBV at 1 month. Differentiated responders and progressive disease.	NA
Quattrocchi et al. 2014^63^	107 (31/76)	LC	T1‐w, T1‐w post, and T2‐w FLAIR	WM hyperintensities alter BMs distribution. Results in unexpected locations compared to the primary tumour.	NA
Almeida_freitas et al. 2014^28^	16	NSCLC	DCE	SRS linked with reduced K^trans^ values.	NA
O'dowd et al. 2014^64^	646	NSCLC, SCLC, adenocarcinoma, and adenosquamous	NA	Preoperative MR brain scan is recommended for Patients with NSCLC undergoing curative surgery. Recommended regardless of preoperative stage, but particularly for those with adenocarcinoma histology. MR and CT brain imaging are superior to detecting small metastases (<1 cm), posterior fossa lesions, and multiple metastases.	NA
Zhong et al. 2015^65^	48 (22/26)	NSCLC	T1‐w, T1‐w post, T2‐w, and T2‐w FLAIR	MRI shows bilateral diffuse WM T2 hyperintensity surrounding periventricular regions in delayed leukoencephalopathy patients U‐fibre, callosum, and grey matter structure are spared.	Median KPS of 90. Onset of symptoms after WBRT: 6 months. Headache reported by 70.8% of patients Cognitive impairment was reported by 41.7% of patients. Motor deficits were reported by 22.9% of patients. Confusion reported by 4.2% of patients Average number of BM lesions at diagnosis: 5.8. BMs improved with WBRT in 60.4% of patients.
Li et al. 2016^23^	126	NSCLC, SCLC, adenocarcinoma, and adenosquamous	T1‐w, T1‐w post, T2‐w, and DWI	TA can differentiate LC from BMs. Textural characteristics reflect tumour histopathological structure. TA has potential as an additional diagnostic tool.	NA
Nardone et al. 2016^66^	38 (31/7)	NSCLC	T1‐w, T1‐w post, and FLAIR	Image‐based technique may aid the clinical decision process for Patients with NSCLC. MRI‐based TA for oligo‐BMs undergoing SRS or SRT outcomes is promising.	Median OS: 3.5 months. Median times to local progression (L‐TTP): 3.5 months. Median new BMs (N‐TTP): 3 months.
Bette et al. 2017^67^	5 out of 41	LC	T2‐w FLAIR	FLAIR signal alterations	23 out of 41 patients experienced disease recurrence after initial treatment. Local tumour recurrence was observed in 6 cases. Distant tumour recurrence was observed in 17 cases. Median observation duration was 462 days.
Yin et al. 2017^68^	69 (51/18)	NSCLC	T1‐w, T1‐w MPRAGE, and T2‐w	11 brain areas' volumes correlated with BM incidence. Change in GM levels can cause psychological problems. GM frontal region linked to patient's mental condition. Olfactory sensations linked to hippocampus, a symptom of NSCLC with BM. Neurological symptoms common in NSCLC with BM, caused by cerebellar lesions affecting the precentral gyrus or basal ganglia. BM development in NSCLC accompanied by aberrant alterations in brain anatomy.	
Wang et al. 2017^29^	68	LC	DCE	Lung carcinoma had high levels of MMP‐9 and VEGF expression. MMP‐9 and VEGF were strongly linked to K^trans^. K^trans^ of peritumoral cerebral edema can assess invasiveness and vascular permeability of BMs in patients with LC.	
Kuchcinski et al. 2017^30^	44 (33/11)	NSCLC and SCLC	DCE	DCE MRI and early ∆ve can predict the objective response of BMs. ve: Extravascular extracellular space per unit volume of tissue	24 patients survived midterm, with a 54.5% OS rate. 18 patients had stable disease or better, resulting in a PFS rate of 40.9%.
Taunk et al. 2018^31^	41 (21/20)	NSCLC	DCE	Post‐treatment Transfer constant (K^trans^) SD predicts the long‐term response of LC BMs to SRS. K^trans^ SD serves as an early imaging biomarker.	Post radiation therapy tumour volume was reduced from 450 to 210 mm^3^.
Liu et al. 2018^38^	60	NSCLC	T1‐w, T1‐w post, T2‐w FLAIR, DWI, and MRS	Island sign present in individuals with longer lifetimes, high signal rings in T2‐w FLAIR, raised lipid peaks (using MRS), and decreased ADC values after radiation for BMs. Cho/Cr >2 or an increase in metastatic size are not accurate predictors of BM progression.	Median survival time of patients: 15.78 months.
Muto et al. 2018^69^	12	LC	DSC	Distinguish tumour recurrence, necrosis, and pseudo‐progression using DSC. rCBV was the most accurate and reliable method.	Clinical outcome was pseudo‐progression (size).
Knitter et al. 2018^32^	13	LC	DSC and DCE	SRS used for BM treatment. Pseudo‐progression vs disease progression differentiation possible. Interval decrease in relative CBV and K^trans^ values used as distinguishing factors.	
Kazda et al. 2018^70^	120	NSCLC, SCLC, and LC	T1‐w Post	50% risk reduction in subsequent recurrence with unilateral hippocampal sparing WBRT.	Quality of life and neurocognitive tests are recommended.
Skogen et al. 2019^71^	5	LC	DTI	Peritumoral extracellular matrix distinguishes GBMs from BMs. extracellular matrix is caused by the infiltrative nature of GBM.	GBMs have higher heterogeneity in peritumoral edema compared to BMs. Sensitivity for identifying this difference is 80%. Specificity for identifying this difference is 90%.
She et al. 2019^72^	15	LC	DSC	rCBV in PBZ is efficient in differentiating GBs from BMs. BMs have higher edema compared to GBs.	
Zhang et al. 2019^39^	17	LC	DWI	ADC‐based TA distinguishes GBM from BMs. ROI on solid portion recommended for generating texture metrics.	NA
Lang et al. 2019^33^	30 (16/14)/56	LC	DCE	DCE‐MRI machine‐learning analysis could Predict spine LC metastases.	NA
Bachmann et al. 2019^73^	37	NSCLC	T1‐w post and T2‐w	Routine MRI follow‐up did not improve OS, symptom‐free survival, WBRT‐free survival, or WBRT deferral. 58 patients had a Graded Prognostic Assessment score >2.5. Median OS was 19.4 months. 16 (21%) patients had neurological death.	
Chakhoyan et al. 2019^40^	43	NSCLC	T1‐w, T1‐w post, T2‐w FLAIR, DWI, and DSC	Independent component analysis‐derived component analysis reveals perfusion differences in metastatic brain tumours, Traditional measures like ADC and rCBV cannot detect these differences,	NA
Morabito et al. 2019^34^	NA (Lung)	LC	DSC and DCE	MRI perfusion methods aid in distinguishing tumour recurrence from radiation necrosis, DCE is a notable perfusion method used for follow‐up after radiosurgery.	NA
Schoenmaekers et al. 2019^35^	149	NSCLC and SCLC	MRI	Although BMs were found in 7% of otherwise stage III Patients with NSCLC using dedicated contrast‐enhanced–computed tomography (dCE‐CT) for staging, MRI of the brain found them in an extra 4.7% of patients, which is clinically significant.	
Chu et al. 2019^74^	110 (95/15)	LS‐SCLC	T1‐w, T1‐w post, and T2‐w	MRI examination necessary before PCI.	Chemoradiotherapy length was identified as a significant risk factor for BMs before PCI in risk analysis.
Borghei‐Razavi et al. 2020^41^	26	LC	DWI	Radiation necrosis prediction requires target volume and ADC knowledge pre‐surgery.	NA
Yang et al. 2020^27^	26 (17/9) ‐ 51.36		T1‐w, T1‐w post, T2‐w, T2‐w FLAIR, DWI, SWI, ASL, and CEST	CEST reflects protein metabolism Molecular imaging can detect BMs early Molecular imaging can evaluate patients' prognosis	NA
Huang et al. 2020^75^	161 (65/96)/61.5	NSCLC	T1‐w post	Karnofsky Performance Scale (KPS) rating: 90 (average). Zone percentage of BMs from pre‐GKRS contrast‐enhanced T1‐w is an independent predictor of local tumour control after GKRS for patients with non‐small cell LC with BMs. Radiomic features reveal the biological foundation and unique properties of tumours. Radiomic features may serve as surrogate biomarkers to predict tumour prognosis after GKRS.	
Wang et al. 2020^76^	50	NSCLC	T1‐w post and T2‐w	Brain MRI performed 1 month after SRS and every 3 months thereafter. T2‐w sequences used to identify perilesional edema. T1 gadolinium‐enhanced images used to define gross tumour volume.	Endpoints: intracranial ORR, intracranial PFS, ORR, disease control rate (DCR), PFS, OS, safety, SRS rate after anlotinib treatment.
Teyateet et al. 2020^77^	34	NSCLC	T1‐w post and T2‐w	T2‐w shows smaller planning target volumes than T1‐w post in most cases. T2‐w accurately delineates the resection cavity even early after surgery.	12‐month OS rate: 76% 24‐month OS rate: 53% Median OS: 25 months
Kim et al. 2020^78^	203	NSCLC	T1‐w post	MRI of the brain has low diagnostic yield in stage IA NSCLC. In stage IB and EGFR mutation‐positive patients, an MRI of the brain has a higher diagnostic yield.	NSCLC yields: 0.3% (2/615) for stage IA, 3.8% (7/186) for stage IB, and 4.7% (8/171) for stage II.
Bozdag et al. 2021^24^	50	NSCLC, SCLC, SCC, and adenocarcinoma	T1‐w post, T2‐w, and DWI	ADC histogram analysis can differentiate between LC‐BMs histological subtypes. ADC25 was found to be the most accurate measure for discriminating NSCLC‐BMs from SCLC‐BMs. This differentiation has high prognostic significance.	NA
Park et al. 2021^42^	51 (26/25)	NSCLC	T1‐w, DWI, and DTI	Radiomics classifiers can help distinguish EGFR mutation status in BMs. Multiparametric MRI parameters are incorporated in the radiomics classifiers. This distinction may be made from NSCLC.	12% of primary tumours and BMs had different EGFR mutation status. Best performing radiomics classifier used 5 features from ADC, FA, and T1‐w images. AUC, accuracy, sensitivity, and specificity were 0.73, 78.6%, 81.3%, and 76.9% in the test set.
Zhao et al. 2021^43^	102 (58/44)	NSCLC	T1‐w, T1‐w post, T2‐w, T2‐w FLAIR, and DWI	ADC value is a promising biomarker for predicting tumour response to WBRT in BMs of patients with NSCLC. ADC value is more useful compared to established imaging assessment methods. Predictions can be made before treatment starts.	NA
Zhao et al. 2021^44^	24 (11/13)	NSCLC	T1‐w, T1‐w post, T2‐w, T2‐w FLAIR, and DWI	MRI‐based radiomics predict PFS and progression in ALK‐positive Patients with NSCLC treated with ensartinib. Enables risk classification and individualised follow‐up and treatment.	Significant difference in PFS between high‐risk and low‐risk groups.
Grossman et al. 2021^79^	69 (41/28)	NSCLC and SCLC	T1‐w post, T2‐w, and FLAIR	DLM: Non‐invasive and automated method. Categorises BMs. Distinguishes NSCLC and SCLC metastases. High sensitivity and specificity.	NA
Alemany et al. 2021^80^	28 (20/8)/55.57	NSCLC	T1‐w post, and FLAIR	F/Gd ratio shows promise for predicting oligosymptomatic patients with multiple BMs. Can potentially delay need for WBRT. Based on the ratio of maximum diameter in FLAIR and T1‐Gd sequences.	NA
Jünger et al. 2021^81^	98	NSCLC	T1‐w, T1‐w post, T2‐w, and T2‐w FLAIR	DLM suggested for BM detection in NSCLC. Better segmentation performance. Higher detection sensitivity.	NA
Kim et al. 2021^82^	72 (39/33)	NSCLC	T1‐w post	Follow‐up MRI of the brain after 12 months of initial diagnosis can monitor high‐risk patients with NSCLC This monitoring can lead to earlier detection and targeted treatment	6, 8, 24, and 34 patients had clinical stages I, II, III, and IV disease 56 patients had adenocarcinoma (including 36 with EGFR mutation‐positive adenocarcinoma and 4 with ALK‐rearranged adenocarcinoma) 11 patients had SCC
Wang et al. 2021^45^	215 (157/58)/61	SCLC	T1‐w, T1‐w post, T2‐w, and T2‐w FLAIR	Cerebellum had high number of SCLC‐BMs BMs rare in SCLC despite critical brain structures.	NA
Wang et al. 2021^26^	52 (26/26)	Adenocarcinoma	T1‐w, T1‐w post, T2‐w FLAIR, and DWI	Non‐invasive radiomics signature created from T2‐FLAIR imaging. Aim is to predict EGFR mutation status in LC.	Mutant group: 15 instances with KPS ≥ 70, 13 instances with KPS < 70. Wild‐type group: 16 instances with KPS ≥ 70, 8 instances with KPS < 70.
Liao et al. 2021^49^	237 (115/122)	NSCLC	T1‐w, T1‐w post, and T2‐w	Median OS of 12.2 months observed. MRI radiomics improves the accuracy and reliability of prediction models for local tumour management and OS. MRI homogeneity and correlation can infer tumour radio‐resistance to some extent. Histogram features, correlation, and cluster tendency from MRIs can enhance OS prediction, along with clinical variables.	
Han et al. 2021^50^	76	LC	T1‐w post and T2‐w	Radiomics analysis improves discriminatory power for GBM, MET‐lung, and MET‐other compared to non‐radiomic analysis. A combination of radiomic and non‐radiomic traits can differentiate between the three tumour types.	
Madamesila et al. 2021^46^	6	LC	T1‐w post and DWI	ADC value changes can be observed in metastases before structural imaging. ADC changes vary before and after therapy in metastases. ADC is similar across metastatic tumours before SRS. Tumours show distinct ADC changes during the first 6 months after SRS.	NA
Jiang et al. 2022^36^	137	LC	T1‐w, T1‐w post, T1‐w MPRAGE, T2‐w, T2‐w FLAIR, DCE, and DWI	Clinical parameters (tumour size and SRS dosage) cannot predict the posttreatment response of LC‐BMs to GKRS. A radiomics method combining MRI‐based radiomics characteristics and clinical variables was developed for accurate prediction. The radiomics method showed high accuracy and reliability in predicting posttreatment response. The method could aid in quicker decision‐making for treatment changes.	
Zheng et al. 2022^83^	162 (97/65)	LC	T1‐w, T1‐w post, T2‐w, and T2‐w FLAIR	84% of BMs show evidence of cysts. 30% of BMs show evidence of haemorrhages. Radiomic signatures incorporating multi‐sequence MR images could predict EGFR mutation status in LC‐BMs non‐invasively.	
Bilgin et al. 2022^84^	146	LC	T1‐w post	BM patients exhibit varying levels and types of vasogenic edema according to tumour location and type. Adenocarcinoma, especially LC, correlates with higher edema‐mass ratio.	NA
Li et al. 2022^51^	186 (73/113)	NSCLC	T1‐w post, T2‐w, and T2‐w FLAIR	Noninvasive diagnostic techniques are valuable for therapeutic techniques. T2‐w FLAIR and T1‐w post‐radiomics models can determine EGFR and ALK mutation status.	
Fan et al. 2022^52^	110 (48/62)	NSCLC	T1‐w post and T2‐w	Both tumour active area and peritumoral edema area on pretreatment brain MRI for NSCLC with BMs could detect T790M resistance mutation. Multi‐region combined radiomics signature may serve as a biomarker for evaluating T790M mutation. High‐risk cancer patients may require more frequent brain MRI monitoring to reduce treatment‐related morbidity.	
Wu et al. 2022^85^	84 (40/44)	LC	T1‐w, T1‐w post, andT2‐w	Prediction model created for BMs probability in LC using clinical and genetic data. Model may improve clinical decision‐making for early‐stage patients with LC at high risk for BMs. Patients with high risk may need more frequent brain MRI monitoring to reduce treatment‐related morbidity.	
Carloni et al. 2023^86^	148 (86/62)/65	NSCLC	T1‐w post	Older age and concurrent treatment increase the chance of complete response in SRS patients. Stage IV cancer and BM diagnosis increase the likelihood of distant progression. Concurrent therapy is protective against distant progression compared to no treatment. Higher biologically effective dose and KPS associated with longer OS. Large amount of tumour within the skull associated with lower OS rate. The efficacy of models may be affected by the radiomic platform used.	
Zheng et al. 2023^47^	48 (29/19)	NSCLC	T1‐w, T1‐w post, T2‐w, T2‐w FLAIR, and DWI	Biomarkers for non‐invasively distinguishing EGFR mutation status in NSCLC BMs. Whole‐lesion ADC histogram analysis and MRI characteristics may provide these biomarkers.	23 patients with primary LC. caused by EGFR mutations. 10 had exon 19 deletion. 12 had exon 21 missense mutations. 1 had exon 18 missense mutation.
Kiyose et al. 2023^48^	38	NSCLC and SCLC	T1‐w, T1‐w post, T2‐w, T2*‐w, T2‐w FLAIR, and DWI	No distinct MRI signal for determining BM origin was identified. Low and high‐proliferative BMs have distinct MRI characteristics and histological profiles. This may enable non‐invasive differentiation of NSCLC and SCLC‐BMs in clinical settings.	
Chu et al. 2023^53^	256 (122/134)	NSCLC	T1‐w	MRI radiomics model evaluated for predicting BMs and guiding patient selection for BMs prevention PCI. The model had an AUC of 0.75 with the training dataset and 0.67 with the testing dataset. Model has potential clinical utility in identifying patients at high risk of BMs who would benefit from prevention PCI while avoiding unnecessary neurocognitive toxicities in those with low risk of BM.	
Sui et al. 2023^87^	192 (123/69)/61	NSCLC and SCLC	T1‐w post	DLM suggested identifying subgroups with pathological changes leading to BMs in SCLC and NSCLC.	NA

Abbreviations: ADC, Apparent diffusion coefficient; ALK, Anaplastic lymphoma kinase; ASL, Arterial spin labeling; BMs, Brain metastases; CBF, Cerebral blood fluid; CBV, Cerebral blood volume; CE, Contrast‐enhanced; CEST, Chemical exchange saturation transfer; CNS, Central nervous system; CT, Computed tomography; DCE, Dynamic contrast‐enhanced; DL, Deep learning; DLM, Deep learning method; DSC, Dynamic susceptibility contrast; DWI, Diffusion‐weighted imaging; EGFR, Epidermal growth factor receptor; FLAIR, Fluid attenuated inversion recovery; GBM, Glioblastoma multiforme; GKRS, Gamma knife radiosurgery; KPS, Karnofsky performance status; Ktrans, Transfer constant; LC, Lung cancer; MRI, Magnetic resonance imaging; MRS, Magnetic resonance spectroscopy; NSCLC, Non‐small cell lung cancer; ORR, Objective response rate; OS, Overall survival; PBZ, Peritumoral brain zone; PCI, Prophylactic cranial irradiation; PFS, Progression‐free survival; PRISMA, Preferred Reporting Items for Systematic Reviews and Meta‐Analyses; PWI, Perfusion‐weighted imaging; rCBV, Relative CBV; ROI, Region of interest; SCLC, Small cell lung cancer; SRS, Stereotactic radiosurgery; SRT, Stereotactic radiotherapy; SWI, Susceptibility‐weighted imaging; T1‐w, T1‐weighted; T1‐w post, T1‐weighted post‐contrast; T2‐w, T2‐weighted; T2‐w FLAIR, T2‐weighted fluid attenuated inversion recovery; WBRT, Whole‐brain radiotherapy; WM, White matter.

The risk of bias in the included studies was evaluated using the Cochrane Risk of Bias tool for randomised controlled trials and the Newcastle–Ottawa Scale for non‐randomised studies. Three reviewers independently assessed the risk of bias for each study, and any disagreements were resolved through discussion and consensus.

**Figure 1:**
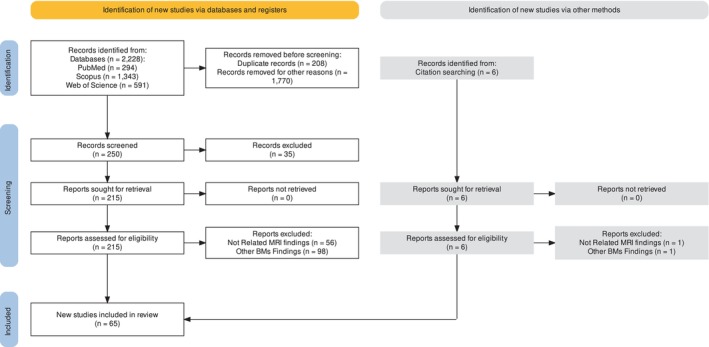
PRISMA flow diagram for systematic review process management.

A total of 2228 studies were identified from the initial search, and duplicates were removed after combining the results from the searches. Studies that did not meet the inclusion criteria were excluded during this phase. The studies were screened based on titles and abstracts, and 1978 studies were excluded. The remaining 250 studies were reviewed in full text, and 185 were excluded based on eligibility criteria. After the second screening, 61 studies met the eligibility criteria and were included in the final analysis. Additionally, four studies were identified through manual searching of the references cited within the included studies. This review identified 65 studies that met the inclusion criteria and provided information on the clinical and technical aspects of LC (Fig. 1).

## Results Overview

This summary presents the results of various studies on LC, non‐small cell lung cancer (NSCLC), small cell lung cancer (SCLC), adenocarcinoma and other subtypes. The studies used different MRI techniques, including T1‐w, T1‐w post, T2‐w, T2‐w FLAIR, DWI, dynamic contrast‐enhanced (DCE) and dynamic susceptibility contrast (DSC). The number of subjects in each study ranged from 5 to 1792, with varying male/female ratios and mean ages. The studies were conducted between 2000 and 2023 by various researchers and institutions.

The 65 articles cover a diverse range of subject populations, tumour properties and MRI techniques, providing a comprehensive overview of the research conducted in this field (Table 1). The studies presented in the table aimed to investigate the role of MRI in the detection, diagnosis and treatment of BMs in patients with LC. The tumour properties of the patients in the studies were mostly limited to NSCLC and SCLC, with some studies including adenocarcinoma, adenosquamous and other unspecified lung tumours.

The subject information, including gender distribution and mean age, varied between studies. Some studies examined multiple tumour types,^23^, ^24^ while others focussed on specific tumour subtypes or single tumour types.^25^, ^26^ In terms of the specific properties investigated, the studies reported a range of characteristics, including tumour size, location and stage. Some studies also examined the relationship between MRI findings and tumour histology, as well as the association between MRI findings and various prognostic factors, such as survival and response to treatment.

A wide range of MRI techniques were employed across the studies. Some commonly used techniques include T1‐w, T1‐w post, T2‐w and T2‐w FLAIR sequences. Advanced MRI techniques, such as DWI, SWI, arterial spin labeling (ASL) and chemical exchange saturation transfer (CEST), were also used in some studies.^27^ Additionally, PWI techniques (including DCE and DSC) were employed in several studies for the characterisation of BM.^28^, ^29^, ^30^, ^31^, ^32^, ^33^, ^34^ The parameters of PWI, including cerebral blood flow (CBF), cerebral blood volume (CBV), relative CBV (rCBV) and transfer constant (K^trans^), showed significant differences between BMs and other intracranial lesions. Moreover, these parameters were found to be useful for evaluating the response to treatment and predicting survival in BM patients.^28^, ^29^, ^31^, ^32^

Several studies in Table 1 investigated the use of DCE‐MRI specifically, which involves administering a contrast agent and measuring the change in signal intensity over time to assess tumour vascularity. The results of these studies suggest that DCE‐MRI can provide valuable information about tumour perfusion and vascularity, which may be useful in predicting treatment response and guiding treatment decisions.^30^, ^31^, ^33^ One notable method for follow‐up after radiosurgery is DCE‐MRI, which provides information about the blood–brain barrier and microvascular permeability. This can be particularly useful in differentiating between tumour recurrence and radiation necrosis, as both conditions can present with similar imaging features such as increased signal intensity in T2‐w or FLAIR images. Studies have shown that DCE‐MRI can help distinguish between these two conditions with a high degree of accuracy.^33^, ^34^, ^35^, ^36^

Diffusion‐weighted imaging was another commonly used MRI technique in the studies. Diffusion‐weighted imaging measures the movement of water molecules in tissues and can provide information on tissue cellularity and integrity. Studies suggest that DWI can be useful to distinguish between histological subtypes of LC and to assess treatment response.^37^, ^38^, ^39^, ^40^, ^41^, ^42^, ^43^, ^44^, ^45^, ^46^, ^47^, ^48^

Radiomics and DLMs demonstrated high accuracy in distinguishing BMs from other brain lesions.^36^, ^42^, ^44^, ^45^, ^49^, ^50^, ^51^, ^52^, ^53^ Additionally, they showed the potential to predict the risk of BMs in patients with NSCLC and SCLC. These models used different radiomic features, such as texture features, shape features and intensity features, to characterise the tumours. The use of DL algorithms improved the specificity of the models, allowing for the identification of specific subgroups of NSCLC and SCLC that have pathological changes leading to BMs.^36^, ^44^, ^45^, ^53^

## Discussion

Brain metastases from LC are a significant source of morbidity and mortality for patients with this disease.^88^, ^89^ The development of BMs is associated with a poor prognosis and a reduced quality of life. As systemic therapy for LC improves and patients live longer, the incidence of BMs is increasing and has become a major impediment to further improvements in survival. Imaging with MRI plays a central role in the screening, diagnosis and follow‐up of BMs. This review aimed to summarise recent MRI advances in the clinical and technical aspects of BMs from LC, including prediction models, prognostic factors, biomarkers and treatment modalities.

### Advanced MRI

Advanced MRI sequences and techniques such as 3D MPRAGE, SWI and PWI methods have shown promise for improved detection and characterisation of BMs in patients with LC.^4^, ^10^, ^90^ Specifically, studies have shown that 3D MPRAGE is more sensitive than 2D MRI to detect BMs,^56^ while SWI is valuable for detecting cerebral microbleeds indicative of metastatic spread.^39^ Perfusion MRI techniques such as DCE and DSC have also emerged as useful tools for differentiating radiation necrosis from tumour recurrence in patients treated with radiosurgery.^32^, ^40^, ^58^, ^62^, ^69^, ^72^ However, questions remain about the optimal timing and frequency of MRI surveillance MRI for BM screening and monitoring.^35^, ^73^, ^85^ Overall, advanced MRI methods have the potential to improve diagnostic accuracy, treatment planning and prognostication for LC patients at risk of or with established BMs, although further research is needed to determine the ideal incorporation of these modalities into clinical practice.

K^trans^ and K^2trans^ are parameters used in DCE‐MRI to measure the transfer of contrast agents from blood plasma to the extravascular extracellular space (EES). K^trans^ represents the volume transfer constant between blood plasma and EES, while K^2trans^ is the volume transfer constant between the slow compartment and EES in the two‐tissue compartment model.^91^, ^92^ Some studies have investigated the use of PWI techniques for predicting treatment response and patient survival outcomes. For example, a study found that lower K^2trans^ at 1 week and rCBV at 1 month can differentiate responders from progressive disease.^62^ Another study found that post‐treatment K^trans^ may predict the response of LC‐BMs to stereotactic radiosurgery (SRS).^31^ These findings suggest that PWI techniques may be a valuable tool for predicting treatment outcomes and guiding personalised therapy for BMs of patients with LC.

In addition, some studies have reported that PWI techniques can assist in the differential diagnosis of tumour recurrence and radiation necrosis after radiosurgery follow‐up.^34^, ^69^ This finding suggests that PWI techniques may be a valuable tool for improving the accuracy of diagnosis and treatment planning for BMs of patients with LC. In summary, PWI techniques, such as DCE and DSC MRI, have emerged as promising tools to improve the accuracy of diagnosis, characterisation and treatment planning for BMs of patients with LC. These techniques may also be valuable for predicting treatment outcomes and guiding personalised therapy. Further research is needed to fully understand the potential of these methods and address the challenges associated with standardisation and validation.

Several studies investigated biomarkers to non‐invasively distinguish EGFR mutation status in BMs from NSCLC. The biomarkers investigated in these studies included whole‐lesion apparent diffusion coefficient (ADC) histogram analysis and MRI characteristics.^42^, ^47^, ^78^, ^82^ One study found that these biomarkers may be used as potential diagnostic tools to distinguish the status of EGFR mutation.^83^

### Machine and deep learning methods

Machine learning models were developed to help identify specific subgroups of SCLC and NSCLC that are more prone to developing BMs.^79^, ^81^, ^87^ One study suggested a DLM that may help identify these subgroups.^87^ These findings may help inform future research on personalised approaches for managing BMs from LC.

In recent years, there has been a growing interest in utilising radiomics and DLM to improve the detection, diagnosis and treatment of BMs. Radiomics is a rapidly evolving field that involves the extraction and analysis of quantitative features from medical images.^79^, ^81^, ^87^ Several studies have shown that radiomic features derived from pre‐treatment T1‐w can serve as surrogate biomarkers to predict local tumour control after gamma knife radiosurgery (GKRS) in non‐small cell BMs of patients with LC.^61^, ^76^ Additionally, radiomics classifiers incorporating multiparametric MRI parameters have been shown to distinguish the EGFR mutation status in BMs from NSCLC, achieving high accuracy, sensitivity and specificity.^47^, ^51^, ^83^ These findings suggest that radiomics may be a valuable tool for predicting treatment outcomes and guiding personalised therapy for BMs of patients with LC.

Several studies have demonstrated the potential of DLM to improve the detection and segmentation of BMs in LC patients. For instance, a suggested DLM has shown better segmentation performance for BM detection in NSCLC.^81^ Another study found that a seed‐and‐soil radiomics model showed promise in distinguishing between high‐risk and low‐risk patients for developing BMs in LC, which could help prevent unnecessary neurocognitive toxicities from prophylactic cranial irradiation (PCI).^79^ These findings suggest that DLM can be a valuable tool for improving the accuracy and efficiency of BM detection and diagnosis.

Several studies have demonstrated the potential of radiomics features extracted from multiparametric MRI and DLM to non‐invasively predict molecular alterations and outcomes in patients with LC BMs. Radiomics signatures have shown promise in predicting the status of EGFR, anaplastic lymphoma kinase (ALK) and T790M mutation status,^42^, ^45^, ^51^, ^52^, ^83^ which could facilitate personalised therapy selection. Radiomic models have also been used to predict the response to treatment after radiosurgery or whole brain radiation,^36^, ^44^, ^75^ OS,^49^ and to differentiate between primary and metastatic brain tumours.^50^, ^79^ These applications underscore the value of radiomics in guiding individualised treatment strategies. Additionally, radiomic models and DL approaches have been proposed to identify LC patients at high risk of developing BMs based on clinical, genetic, and imaging data.^53^, ^85^, ^87^ Such prediction models could optimise brain imaging surveillance and early intervention in high‐risk subgroups.^53^, ^85^ To sum up, radiomics and DL applied to neuroimaging data hold substantial promise for personalised medicine and improved outcomes in BMs of patients with LC.

Texture analysis (TA) was identified as a potential diagnostic tool to distinguish between different pathogenic forms of LC with BMs,^23^ and MRI‐based TA showed promise in clinical decision‐making for patients with NSCLC undergoing SRS or stereotactic radiotherapy (SRT).^66^ The ability to differentiate between various types of BMs is critical in determining the most appropriate treatment strategy. Some studies revealed specific MRI characteristics that can help distinguish between SCLC metastases, glioblastomas, and NSCLC metastases.^24^, ^37^, ^71^ Furthermore, one study identified distinct MRI and histological profiles that may allow for non‐invasive differentiation between NSCLC‐BMs and SCLC‐BMs in typical clinical settings.^48^

### Treatment response and survival outcomes

Radiation therapy modalities such as SRS and whole brain radiation therapy (WBRT) have been evaluated for managing BMs,^43^, ^60^, ^61^, ^65^, ^70^, ^73^, ^80^ but more research is needed to optimise patient‐specific treatment regimens. OS remains poor, with median survival times of around 9–24 months reported in multiple studies.^54^, ^55^, ^60^ However, one study also noted that measuring volumetric white matter (WM) after WBRT is important in assessing cognitive and functional decline, which can significantly impact the quality of life of these patients.^60^ Early detection and accurate diagnosis of BMs is critical, as the presence of BMs alone can significantly impact survival, regardless of disease extent or symptoms.^54^ Identification of prognostic factors and treatment responses that influence OS outcomes will be key to improving prognosis. While various therapeutic approaches have been investigated, BMs of patients with LC continue to have a decreased quality of life and survival compared to those without BMs. More research into personalised and combinatorial treatment strategies is warranted.

In terms of treatment response evaluation, MRI plays a vital role in assessing the effectiveness of various interventions, such as SRS, WBRT and PCI.^74^, ^76^, ^77^ In particular, ADC changes in metastases can be observed earlier than structural imaging,^46^ and whole‐lesion ADC histogram analysis may provide biomarkers to non‐invasively distinguish the status of the EGFR mutation.^83^ Moreover, brain volume changes have been associated with the incidence of BMs in Patients with NSCLC,^68^ highlighting the importance of MRI in monitoring disease progression.

Other studies investigated the factors associated with poor outcomes in patients with BMs from LC. Prognostic factors identified in these studies included diagnosis time, tumour burden and treatment regimens. One study found that patients diagnosed with BMs at the outset, having a large amount of tumour within the skull, or not receiving any treatment at all were associated with poorer outcomes.^86^ Concurrent therapy, a greater biologically effective dose, and a higher Karnofsky performance status (KPS) were associated with longer OS.^45^, ^65^, ^75^, ^86^ Another study discovered that MR screening of the brain for metastases may improve post‐operative survival in patients with operable lung adenocarcinoma. The study reported that 57% of the patients identified with recurrence after full resection of primary lung adenocarcinoma had BM.^25^ This finding suggests that early detection and treatment of BMs may improve OS outcomes for patients with LC.

A study discovered that the island sign in BMs after radiation is associated with longer lifetimes, high signal rings in T2‐w FLAIR, raised lipid peaks (using MRS), and decreased ADC values. The study reported a median survival time of 15.78 months for BMs of patients with LC.^38^ This finding suggests that radiological markers may be useful to predict survival outcomes for BMs in patients with LC. In total, survival outcomes for BMs of patients with LC are affected by several factors, including the presence of BMs alone, the accuracy of detection and diagnosis, and the choice of treatment modality. Early detection and accurate diagnosis of BM, as well as the use of appropriate treatment modalities, may improve survival outcomes and quality of life for these patients. Radiological markers also offer a promising avenue for predicting survival outcomes and personalised treatment for BMs of patients with LC. More research is needed to fully understand the impact of these factors on survival outcomes and inform clinical decision‐making.

### Consideration points and limitations

Our work offers novel insights into the clinical and technical aspects of BMs from LC. Developing prediction models, identifying prognostic factors, and identifying biomarkers for non‐invasive diagnosis and classification may lead to improved patient outcomes and better treatment decisions. It is essential to validate these findings and develop more precise and personalised approaches to managing BMs from LC. Radiomics and DLM, in combination with PWI techniques, demonstrate significant potential for BMs' diagnosis and management. These quantitative imaging methods can help accurately differentiate BMs from other lesions and predict the risk of BMs in patients with NSCLC and SCLC. Additionally, PWI parameters are useful in evaluating treatment response and predicting survival outcomes. Further research must validate these findings and enhance the models' accuracy.

This review highlighted several limitations of the current literature on MRI detection and characterisation of BMs in LC patients. The studies had heterogeneous subject populations and LC subtypes, and variability in MRI techniques. Furthermore, there is a need to standardise radiomic and DLMs and determine the optimal timing for MRI screening. Large prospective multicenter studies are recommended to validate advanced MRI modalities, with cost‐effectiveness analyses to determine the best approaches to address these limitations. Efforts to standardise radiomic feature extraction and DLM development are also needed. Future research should focus on validating prognostic models that incorporate clinical and imaging features to guide individualised treatment, evaluating combinatorial therapies to improve survival, and determining the ideal frequency of MRI screening for BMs. Standardised multi‐institutional collaborative efforts are key to developing robust radiomic signatures and DL algorithms.

## Conclusion

In conclusion, this review emphasises the emerging role of quantitative MRI techniques for the treatment of BMs in LC. The integration of advanced imaging techniques, molecular profiling, radiomics and DLM offers new opportunities to improve the diagnosis, prognosis and management of BMs from LC. MRI techniques commonly used in studies include standard sequences such as T1‐weighted, T2‐weighted and FLAIR as well as advanced modalities such as DWI, PWI and radiomics analysis. Key findings demonstrate the utility of MRI for distinguishing between primary and metastatic brain tumours, evaluating treatment response, predicting outcomes and informing prognostic models. Advanced MRI methods such as PWI show promise for differentiating tumour recurrence from radiation necrosis, while diffusion imaging can detect early treatment response. Radiomics and machine learning applied to multiparametric MRI data may predict the status and risk of BMs. OS remains poor in BMs in patients with LC, underscoring the need for optimised detection and treatment approaches.

## Funding Statement

This research work was conducted without any external funding. All expenses related to the research were covered by the authors themselves, and no financial assistance or support was received from any funding agency, organisation or institution.

## Author Contributions

S.Gh. and S.M. contributed to the conception and design of the study; S.Gh., M.M., S.M., Z.N.A.P., M.H., R.Kh. and R.Z. contributed to the data collection; and S.Gh., S.M., contributed to the drafting of the text. S.Gh. and S.M. revised all sections. The final version was approved by all authors.

## Conflict of Interest

The authors declare no financial or other conflicts of interest.

## Data Availability

The data that support the findings of this study are available upon request from the corresponding author.
